# The host DHX29 RNA helicase regulates HCMV immediate-early protein synthesis

**DOI:** 10.1128/mbio.03542-25

**Published:** 2026-06-09

**Authors:** Erik M. Lenarcic, Nathaniel J. Moorman

**Affiliations:** 1 Department of Microbiology and Immunology, Lineberger Comprehensive Cancer Center, University of North Carolina at Chapel Hill 318275, Chapel Hill, North Carolina, USA; Princeton University, Princeton, New Jersey, USA

**Keywords:** human herpesvirus, human cytomegalovirus, HCMV, RNA helicase, RNA binding proteins, mRNA translation, protein synthesis, major immediate early gene, lytic replication

## Abstract

The DEAD-box helicase DHX29 plays a critical role in the translation of mRNAs containing complex RNA secondary structure in their 5′ untranslated regions (UTRs). The human cytomegalovirus (HCMV) genome has a high GC content, suggesting that the 5′ UTRs of viral mRNAs may contain significant secondary structure and require DHX29 for efficient translation. We found that depleting DHX29 from primary human fibroblasts prior to infection reduced viral mRNA and protein levels and decreased HCMV replication. The defect in HCMV replication correlated with decreased expression of the HCMV immediate-early proteins IE1 and IE2, which are necessary for the establishment of lytic infection. Analysis of polysome-associated mRNAs revealed that the defect in IE1 and IE2 expression is due to decreased mRNA translation efficiency. DHX29 depletion led to reduced levels of the eIF4F translation initiation complex, resulting from decreased translation of the eIF4G1 mRNA. However, in line with our previous results showing a minimal role for the eIF4F complex in viral mRNA translation, we found that depleting eIF4G prior to infection did not impact IE1 and IE2 translation. Together, our results define a new role for DHX29 in regulating eIF4F-dependent translation and identify a critical role for DHX29 in the translation of HCMV mRNAs.

**IMPORTANCE:**

Expression of the human cytomegalovirus (HCMV) immediate-early proteins IE1 and IE2 is critical for the establishment of lytic replication and the reactivation of latent HCMV infections. Defining the mechanisms controlling HCMV IE1 and IE2 protein expression has the potential to identify new strategies for therapeutic interventions that can limit HCMV disease in immune-naïve and immune-compromised individuals. Our finding that the cellular DHX29 helicase is necessary for the efficient translation of mRNAs encoding IE1 and IE2 suggests that therapies that inhibit DHX29 could potentially be useful in treating HCMV disease and adds to the growing body of literature suggesting that DHX29 activity is a disease driver in multiple indications, including viral disease, inflammation, and cancer.

## INTRODUCTION

Human cytomegalovirus (HCMV) is a significant cause of human disease, with over 60% of adults acquiring a lifelong HCMV infection by adulthood. In healthy adults, latent HCMV infection typically remains asymptomatic; however, decreased immune function allows HCMV to reactivate and spread throughout the host, causing significant morbidity and mortality. In addition, congenital HCMV infection can lead to developmental abnormalities and is a leading cause of birth defects. Despite the high disease burden, there is no approved vaccine for preventing HCMV disease, and current therapies are limited due to associated toxicities and resistance. A better understanding of the HCMV replicative cycle is needed to fill this unmet need by validating novel targets for new antiviral therapies.

Like all viruses, HCMV relies on cellular translation machinery for the expression of viral proteins. While most viruses shut off host protein synthesis to facilitate the expression of viral proteins, HCMV is somewhat unique in that cellular protein synthesis is maintained, and even increased, throughout infection (1). This, in part, reflects the increase in the expression and activity of the host eIF4F translation initiation complex, which regulates the translation of the majority of cellular mRNAs via interaction with the mRNA 5′ untranslated region (5′ UTR) (2). eIF4F-dependent translation initiation begins with recognition of the mRNA 5′ 7-methylguanosine (m^7^G) cap by the eIF4E protein, which in turn recruits the eIF4G scaffold and the eIF4A DEAD-box RNA helicase to form the eIF4F complex on the 5′ end of the mRNA (3). eIF4A unwinds the RNA structure in the 5′ UTR to create a landing site for the remaining members of the preinitiation complex: the 40S small ribosomal subunit, ternary complex, eIF3, eIF5, eIF1, and eIF1A. The preinitiation complex then scans the 5′ UTR until reaching the translation start codon, where the 60S large ribosomal subunit joins to form the mature 80S ribosome, and translation elongation begins. During the initiation stage, the eIF4A helicase moves along the RNA, unwinding secondary structures that would otherwise prevent the preinitiation complex from scanning the 5′ UTR. However, some 5′ UTRs contain extensive RNA secondary structures, which are too stable for eIF4A to unwind. Such mRNAs require additional factors for their efficient translation, including DHX29 (2–4).

DHX29 interacts with the 40S ribosomal subunit and the eIF3 subunit of the preinitiation complex (5–7). On highly structured 5′ UTRs, DHX29 enhances the ability of the preinitiation complex to locate the appropriate initiation codon, even when the start codon is embedded in RNA structure (7, 8). The precise mechanism by which DHX29 facilitates translation of mRNAs containing complex 5′ UTRs is unknown. DHX29 is non-processive and therefore likely does not assist eIF4A in unwinding 5′ UTR RNA structure during initiation complex scanning. Rather, DHX29 binds the 40S ribosomal subunit and changes the way RNA secondary structure interacts with ribosomes (7), suggesting that DHX29 locally unwinds RNA structures proximal to the start site to allow for correct start site recognition.

While DHX29 plays a critical role in the translation of RNA virus genomes, its potential role in the translation of DNA virus mRNAs has not been explored. The HCMV genome has a GC content of 60% (9), increasing the likelihood that the 5′ UTR of any given HCMV mRNA has significant RNA secondary structure. We previously found that neither the eIF4F complex nor the eIF4A RNA helicase are required for efficient HCMV mRNA translation (10), suggesting that additional cellular and/or viral factors are needed to resolve HCMV mRNA 5′ UTR structure to ensure efficient viral protein synthesis. Here, we show that DHX29 is necessary for efficient HCMV lytic replication and the translation of viral mRNAs encoding the IE1 and IE2 proteins. We also show that DHX29 regulates eIF4F-dependent translation by facilitating efficient eIF4G expression. However, the translation of transcripts encoding IE1 and IE2 mRNA occurs independently of eIF4G, providing further evidence that HCMV mRNA translation uses a combination of canonical and non-canonical translation initiation factors to regulate viral protein synthesis.

## MATERIALS AND METHODS

### Cells, plasmids, and viruses

Lentivirus was produced by transfecting HEK 293T cells with either a vector encoding a scrambled control shRNA or a DHX29-specific shRNA together with MISSION Lentiviral Packaging Mix (Sigma) using PEI as before (10, 11). MRC5 primary human fibroblasts were transduced with lentiviruses in the presence of 8 µg/mL polybrene (hexadimethrine bromide, Sigma) in Dulbecco’s Modified Eagle Medium (DMEM, Sigma) with 10% FBS and incubated at 37°C and 5% CO_2_. Seventy-two hours after addition of lentivirus, the cells were either mock-infected or infected with the BAD*in*GFP laboratory strain of HCMV (12) in DMEM with 10% FBS and incubated at 37°C and 5% CO_2_. Cell-free infectious virus production during infection was quantified using a 50% tissue culture infectious dose (TCID_50_) assay as described previously (13). Briefly, 10-fold dilutions of virus were added to MRC5 fibroblasts, with each dilution added to 12 wells of a 96-well tissue culture dish. Fourteen days after the addition of virus, the cells were analyzed for GFP expression using an ultraviolet light microscope (Olympus CKX41 Inverted Phase Contrast Microscope). The number of GFP-positive wells per dilution, as well as the dilution factor, was used to calculate the TCID_50_.

### Sucrose gradient centrifugation and polysome fractionation

Analysis of polysomes was performed as described previously (10, 11, 14, 15). Briefly, cells were incubated with 100 µg/mL cycloheximide (CHX) at 37°C and 5% CO_2_ for 10 min in DMEM and then washed twice with PBS containing 100 µg/mL CHX. The cells were then harvested by scraping into PBS plus CHX, followed by centrifugation at 1,000 × *g* for 10 min. Cell pellets were resuspended in polysome gradient lysis buffer (20 mM Tris-HCl, pH 7.4, 140 mM KCl, 5 mM MgCl_2_, 1% Triton X-100, 100 µg/mL CHX) and allowed to lyse on ice for 10 min. The cells were then sheared by passage through a 27-gauge needle five times, and nuclei were removed by centrifugation at 1,100 × *g* for 5 min. The mitochondria and insoluble material were then removed by centrifugation at 21,000 × *g* for 10 min. The resultant post-mitochondrial supernatants were overlaid onto 10%–50% linear sucrose gradients and centrifuged at 130,000 × *g* for 2 h. The gradient was then fractionated with continuous OD254 monitoring using a UA-6 Absorbance Detector (Teledyne ISCO) and monitored by Peak Chart software (Brandel). To measure transcript abundance in gradient fractions, RNA was extracted as described below and quantified by qRT-PCR. To analyze proteins present in gradient fractions, a 200 µL aliquot of each gradient fraction was mixed with 1 mL of 20% trichloroacetic acid to precipitate proteins, which were then recovered by centrifugation at 21,000 × *g* for 30 min. Protein pellets were washed with acetone, resuspended in protein sample buffer (0.1 M Tris-HCl, pH 6.8, 6% glycerol, 2% SDS, 0.1 M DTT, 0.002% bromophenol blue), resolved by SDS-PAGE, and analyzed by Western blot.

### Quantitative reverse transcriptase PCR (qRT-PCR)

Quantification of specific RNAs was performed essentially as described previously (10, 11, 14, 15). Briefly, RNA was extracted from 200 µL aliquots of individual sucrose gradient fractions using Trizol (Sigma) followed by Turbo DNase (Thermo Fisher) treatment. An equal volume of DNase-treated RNA from each sucrose gradient fraction was used to prepare cDNA using the High Capacity cDNA Reverse Transcription Kit (Thermo Fisher) according to the manufacturer’s directions, and the cDNAs were diluted 1:1 with deionized water prior to PCR. Primers for the following mRNAs were used: IE1 (CAAGTGACCGAGGATTGCAA, CACCATGTCCACTCGAACCTT), IE2 (TGACCGAGGATTGCAACGA, CGGCATGATTGACAGCCTG), β-actin (GACCCAGATCATGTTTGAGACC, GTCACCGGAGTCCATCACGA), eIF4G1 (GCCATTTCAGAGCCCAACTTCTC, CGGAAGTTCACAGTCACTGTTGG), HSP90 (AGATTCCACTAACCGACGCC, CCGCACTCGCTCCACAAA), and RACK1 (TGGGATCTCACAACGGGCACCA, CCGGTTGTCAGAGGAGAAGGCCA). qPCR was performed using SYBR Select Master Mix (Thermo Fisher) and 40 cycles (45 s at 60°C followed by 15 s at 95°C) in a CFX96 thermal cycler (Bio-Rad). Total RNA was prepared and analyzed as above except that 2 µg of RNA was used to prepare cDNA instead of equal volumes. RNA abundance was determined by comparison to a standard curve spanning a range of 10^1^ to 10^8^ amplicon copies using the absolute quantification method.

### Protein analysis

Western blot analysis of protein expression was performed essentially as described previously (10, 11). Cells were harvested by scraping and pelleted by centrifugation at 21,000 × *g* for 10 s. Cell pellets were resuspended in RIPA buffer (50 mM Tris-HCl, pH 7.4, 1% NP-40, 0.25% sodium deoxycholate, 150 mM NaCl, 1 mM EDTA) containing protease inhibitors (Complete EDTA-free protease inhibitor; Roche) and allowed to lyse on ice for 10 min. Insoluble material was removed by centrifugation at 21,000 × *g* for 10 min at 4°C, and the protein concentration in the supernatant was determined by the Bradford assay (VWR). Thirty micrograms per sample was resolved by SDS-PAGE gel electrophoresis and transferred to Protran nitrocellulose membranes (Amersham). Membranes were probed with primary antibodies to DHX29 (Santa Cruz Biotechnology), eIF4E (Cell Signaling Technology), β-actin (Santa Cruz Biotechnology), rpS6 (Cell Signaling Technology), rpL13a (Cell Signaling Technology), HCMV IE1, IE2 (Sigma), UL44/ICP36 (Virusys), UL99 (pp28), eIF4G1 (Cell Signaling Technology), 4E-BP (Cell Signaling Technology), 4E-BP-phospho (Cell Signaling Technology), rpS6-phospho (Cell Signaling Technology), and HSP90 (Stressgen). After probing the membranes with secondary anti-mouse or anti-rabbit antibodies conjugated to HRP (Seracare), bands were detected using WesternBright Enhanced Chemiluminescence (ECL; Advansta) reagents and a ChemiDoc MP Imaging System (Bio-Rad). Protein band densitometry was performed using Image Lab software (Bio-Rad).

### Cap-binding complex precipitation

Isolation of m^7^G associated proteins was performed essentially as described previously (10, 16). Cell pellets were resuspended in CAP IP buffer (40 mM HEPES, pH 7.6, 120 mM NaCl, 1 mM EDTA, 0.3% CHAPS, Complete EDTA-free protease inhibitor) and allowed to lyse on ice for 10 min; then insoluble material was removed by centrifugation at 21,000 × *g* for 10 min at 4°C. The protein concentration of each supernatant was analyzed by Bradford, and equal amounts of protein per condition were used in each experiment. Some samples were incubated with 100 µM soluble 7-methylguanosine 5′-triphosphate (Sigma) for 1 h at 4°C with nutation. Samples were then mixed with immobilized γ-aminophenyl-m^7^GTP agarose beads (Jena Bioscience) and incubated with nutation for 1 h at 4°C. The beads were pelleted by centrifugation at 6,000 × *g* for 1 s and washed with CAP IP buffer three times. The beads were then resuspended in protein sample buffer and boiled at 100°C for 10 min. The supernatants were then separated by SDS-PAGE and analyzed by Western blot.

### Metabolic ^35^S-labeling and immunoprecipitation

Cells were incubated in growth media lacking methionine and cysteine (Sigma) for 15 min at 37°C and 5% CO_2_, then 125 µCi of EasyTag Express^35^S Protein Labeling Mix (Revvity) was added per milliliter of growth media, and the cells were incubated at 37°C and 5% CO_2_ for an additional 30 min. The cells were then washed twice with PBS and harvested, followed by lysis in RIPA buffer. Lysates were pre-cleared prior to immunoprecipitation by incubation for 30 min at 4°C with Protein A/G PLUS-Agarose beads (Santa Cruz Biotechnology) with constant mixing. Then HCMV IE1 protein was precipitated from pre-cleared lysates by incubation for 1 h at 4°C with IE1 antibody pre-conjugated to protein A/G beads with constant mixing. The pre-conjugated beads were then washed three times with RIPA buffer before being resuspended in sample buffer and boiled at 100°C for 10 min. Proteins eluted from the beads were resolved by SDS-PAGE and analyzed by autoradiography (GeneMate).

## RESULTS

### HCMV infection increases the association of DHX29 with the translation machinery during infection

**Fig 1:**
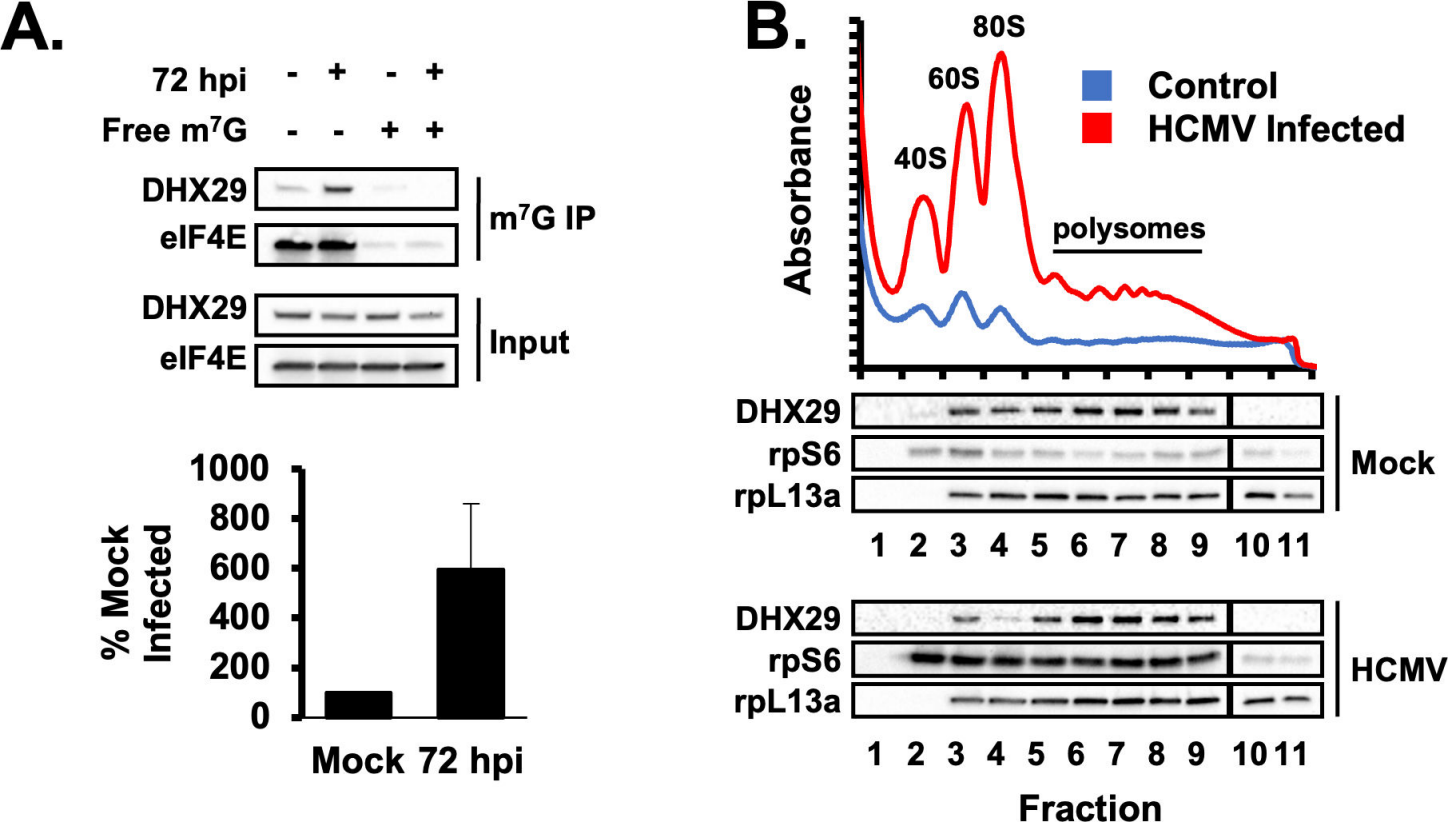
DHX29 associates with the mRNA cap and actively translating mRNAs during HCMV infection in MRC5 primary fibroblasts. (**A**) Top: proteins co-purifying with m^7^G Sepharose (m^7^G IP) or input lysates (input) were analyzed by Western blot at 72 h after HCMV infection (MOI of 3). Excess free m^7^G was included in some reactions (free m^7^G) to control for specificity. Bottom: densitometry of Western blot images was used to calculate the percent change in DHX29 association with m^7^G Sepharose from mock and infected lysates. (**B**) Cytoplasmic lysates from uninfected (mock, blue line) or HCMV-infected (72 hpi, MOI of 3, red line) fibroblasts were resolved through 10%–50% linear sucrose density gradients to separate ribosomal subunits (40S, 60S), single ribosomes (80S), and polysomes. Proteins extracted from each gradient fraction were analyzed by Western blot. Multiple blots prepared and analyzed at the same time were required per gradient, with fractions 1 through 9 on one blot and fractions 10 and 11 on a second blot. The results are representative of at least three independent experiments.

Viruses, including HCMV, manipulate initiation factors during lytic infection to enhance viral mRNA translation (17–23). The RNA helicase DHX29 participates in translation initiation in uninfected cells (24), though its role in translation during HCMV infection is unknown. To determine if DHX29 associates with proteins involved in translation initiation during infection, we measured the association of DHX29 with m^7^G Sepharose in extracts of HCMV-infected cells. Similar levels of eIF4E, the 5′ m^7^G cap-binding protein, co-purified with m^7^G beads incubated with lysates of uninfected and infected cells (Fig. 1A). However, HCMV infection increased the amount of DHX29 associated with m^7^G beads despite similar overall levels of DHX29 in uninfected and infected cell lysates. DHX29 association with m^7^G beads was specific, as the inclusion of excess soluble m^7^G inhibited binding of both eIF4E and DHX29 to the m^7^G beads. These results suggest that HCMV infection increases the association of DHX29 with the mRNA cap or cap-binding complexes.

We also measured the association of DHX29 with actively translating mRNAs during infection by measuring DHX29 association with polysomes in sucrose density gradients. As before, we found that HCMV infection increases the abundance of polysomes as compared to uninfected cells (Fig. 1B), consistent with the global increase in protein synthesis caused by HCMV infection (17, 18, 25–30). DHX29 was detected in fractions containing ribosomal subunits, monosomes, and polysomes in both uninfected and infected cytoplasmic extracts. However, DHX29 was enriched in fractions containing polysomes in extracts from infected cells. Together with the increased association of DHX29 with the m^7^G cap or cap-associated complexes, these results suggest an enhanced role for DHX29 in mRNA translation during HCMV infection.

### DHX29 is required for efficient HCMV amplification

**Fig 2:**
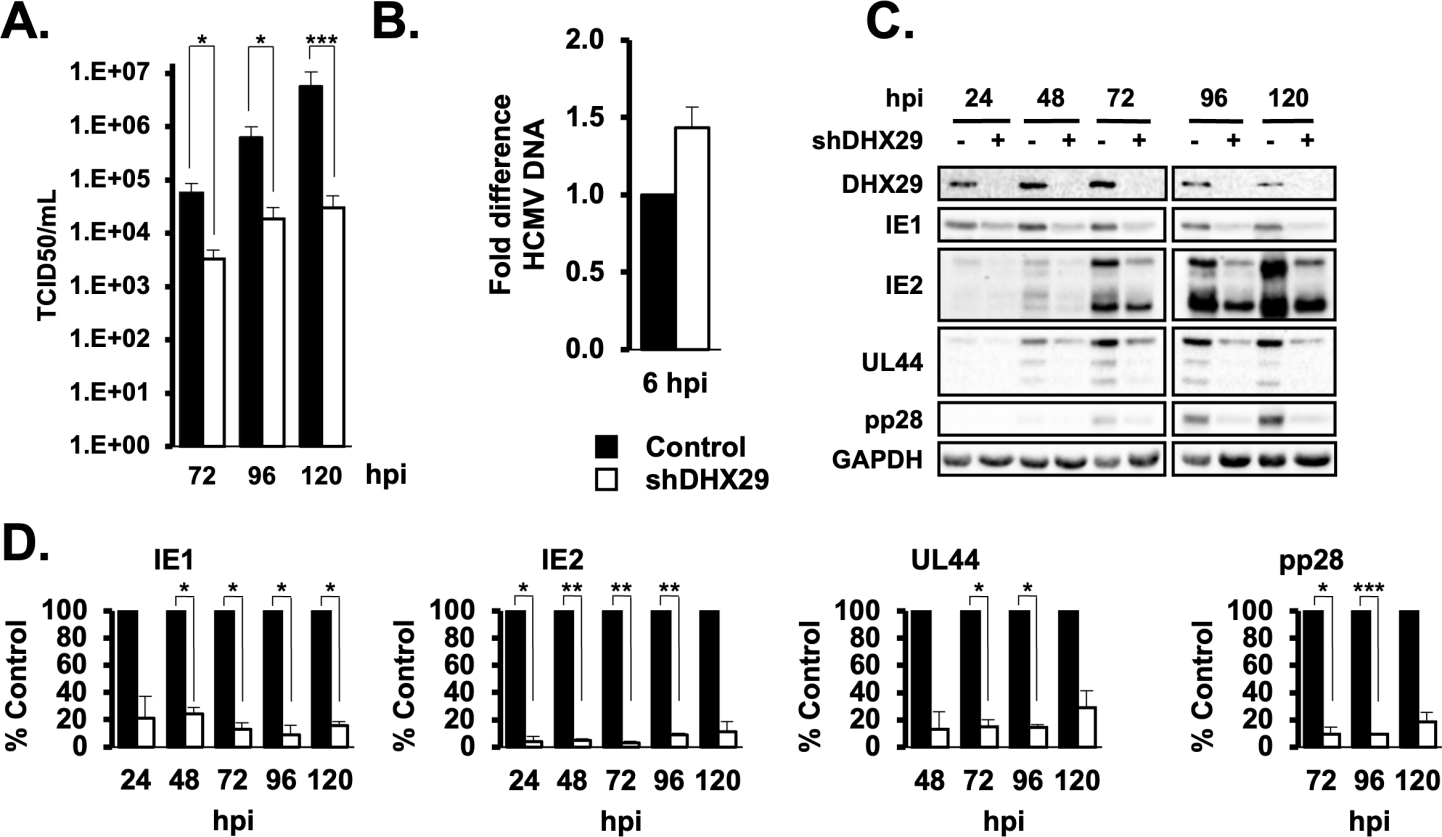
DHX29 is necessary for efficient HCMV replication. (**A**) Human fibroblasts were transduced with lentivirus encoding scrambled (control) or DHX29-specific shRNAs. Seventy-two hours later, cells were infected with HCMV (MOI of 3). Cell-free virus was harvested at the indicated times after infection and quantified by the TCID_50_ assay. (**B**) Cells were transduced and infected as in panel A. The amount of HCMV DNA in infected cells was measured at 6 h after infection by qPCR. The mean data from three independent experiments are shown. (**C**) Cells were transduced and infected as in panel A, and viral protein expression was measured by Western blot at the indicated times after infection. (**D**) Densitometry of Western blot images was used to calculate the percent change in viral protein levels at each time point between control and shRNA-treated cells. The data are representative of at least three independent experiments (*P < 0.05; **P < 0.005; ***P < 0.001).

The increased association of DHX29 with translation initiation complexes and translating mRNAs suggested a potential role for DHX29 in HCMV replication. To determine the role of DHX29 in HCMV replication, we depleted DHX29 by transducing human fibroblasts with lentivirus expressing DHX29-specific shRNAs, which significantly reduced DHX29 protein levels (Fig. 2C). DHX29 did not play a role in virus entry, as equivalent levels of HCMV DNA were observed in control and DHX29-depleted cells at 6 h after infection (Fig. 2B). We then measured the effect on the production of infectious HCMV virions and found that DHX29 depletion decreased the amount of infectious HCMV produced by 17-fold at 72 h, 33-fold at 96 h, and 187-fold at 120 h after infection (Fig. 2A) compared to cells expressing a non-specific control shRNA. Similar effects were observed with a second DHX29-specific shRNA, further confirming the specificity of the result (Fig. S1). The defect in virus replication correlated with delayed and reduced expression of viral immediate-early (IE1, IE2), early (UL44), and late (pp28) proteins throughout the replication cycle (Fig. 2C). Together, these data show that DHX29 expression is critical for efficient viral protein expression and, as a result, HCMV replication.

### DHX29 is necessary for the efficient translation of HCMV immediate-early mRNAs

**Fig 3:**
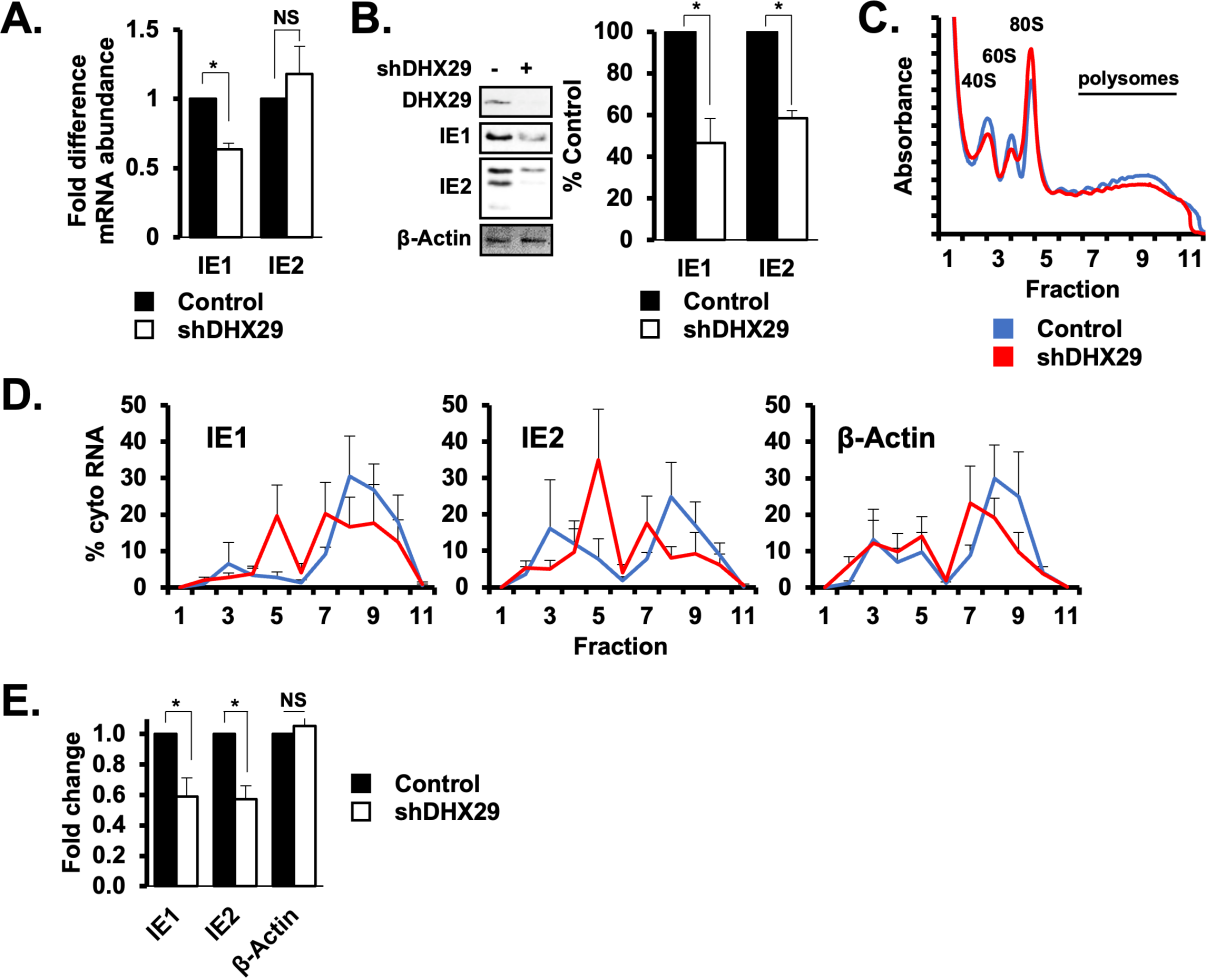
DHX29 is necessary for the efficient translation of mRNAs encoding HCMV immediate-early proteins. Human fibroblasts transduced with lentivirus encoding scrambled control or DHX29-specific shRNAs were infected with HCMV (MOI of 3). Six hours post-infection, HCMV immediate-early mRNA (**A**) and protein levels (**B**, left) were analyzed by qRT-PCR and Western blot, respectively. (**B**, right) Densitometry of Western blot images was used to calculate the percent change in viral protein levels at 6 h post-infection between control and shRNA-treated cells. (**C**) Polysome formation in control or DHX29-depleted cells was measured by sucrose gradient centrifugation at 6 h after HCMV infection. (**D**) The percent of cytosolic RNA found in each gradient fraction was measured by qRT-PCR for the IE1, IE2, and β-actin mRNA. (**E**) Graph of data in panel D, comparing the amount of each mRNA found in gradient fractions containing polysomes in control or DHX29-depleted cells. The amount of each mRNA in control cells was set to 1. The mean of data from three independent experiments is shown (*P < 0.05; **P < 0.005; ***P < 0.001).

The decrease in immediate-early protein expression suggested that DHX29 is required for a very early step in the HCMV infectious cycle. DHX29 was not required for efficient HCMV entry, as similar numbers of viral genomes were found in infected control or DHX29-depleted fibroblasts at 6 h after infection (Fig. 2B). We first measured the effect of DHX29 depletion on the expression of the immediate-early IE1 and IE2 genes. DHX29 depletion led to a modest 40% reduction in IE1 mRNA levels but had no effect on IE2 transcript abundance (Fig. 3A), though the expression of both IE1 and IE2 proteins was decreased when DHX29 was depleted (Fig. 3B).

These results suggested a potential role for DHX29 in regulating the translation of immediate-early HCMV transcripts. To determine if DHX29 was necessary for efficient HCMV IE mRNA translation, we measured the amount of IE1 and IE2 mRNA associated with polysomes in control or DHX29-depleted fibroblasts. Cytoplasmic lysates from HCMV-infected cells were resolved through linear sucrose gradients to measure the abundance of ribosomal subunits, ribosomes, and polysomes in the presence of DHX29. Depleting DHX29 led to a modest increase in the 80S monosome peak and a slight reduction in heavy polysomes at 6 h after infection, consistent with prior studies that found that DHX29 regulates polysome abundance, though the effects of DHX29 depletion were muted compared to prior studies, potentially reflecting cell type-specific roles for DHX29 in translation, indicating that DHX29 depletion did not affect overall levels of translation (Fig. 3C). However, both the IE1 and IE2 mRNAs were less abundant in gradient fractions containing polysomes and more abundant in fractions containing monosomes when DHX29 levels were reduced (Fig. 3D). The amount of IE1 and IE2 mRNAs associated with polysomes was consistently reduced by 50% in DHX29-depleted cells (Fig. 3E). In contrast, DHX29 depletion did not affect the distribution of the host β-actin mRNA in the gradient, and the amount of β-actin mRNA associated with polysomes was unchanged (Fig. 3D and E). These data show that DHX29 is required for the efficient translation of the HCMV IE1 and IE2 mRNAs.

### DHX29 depletion inhibits eIF4G-dependent translation

**Fig 4:**
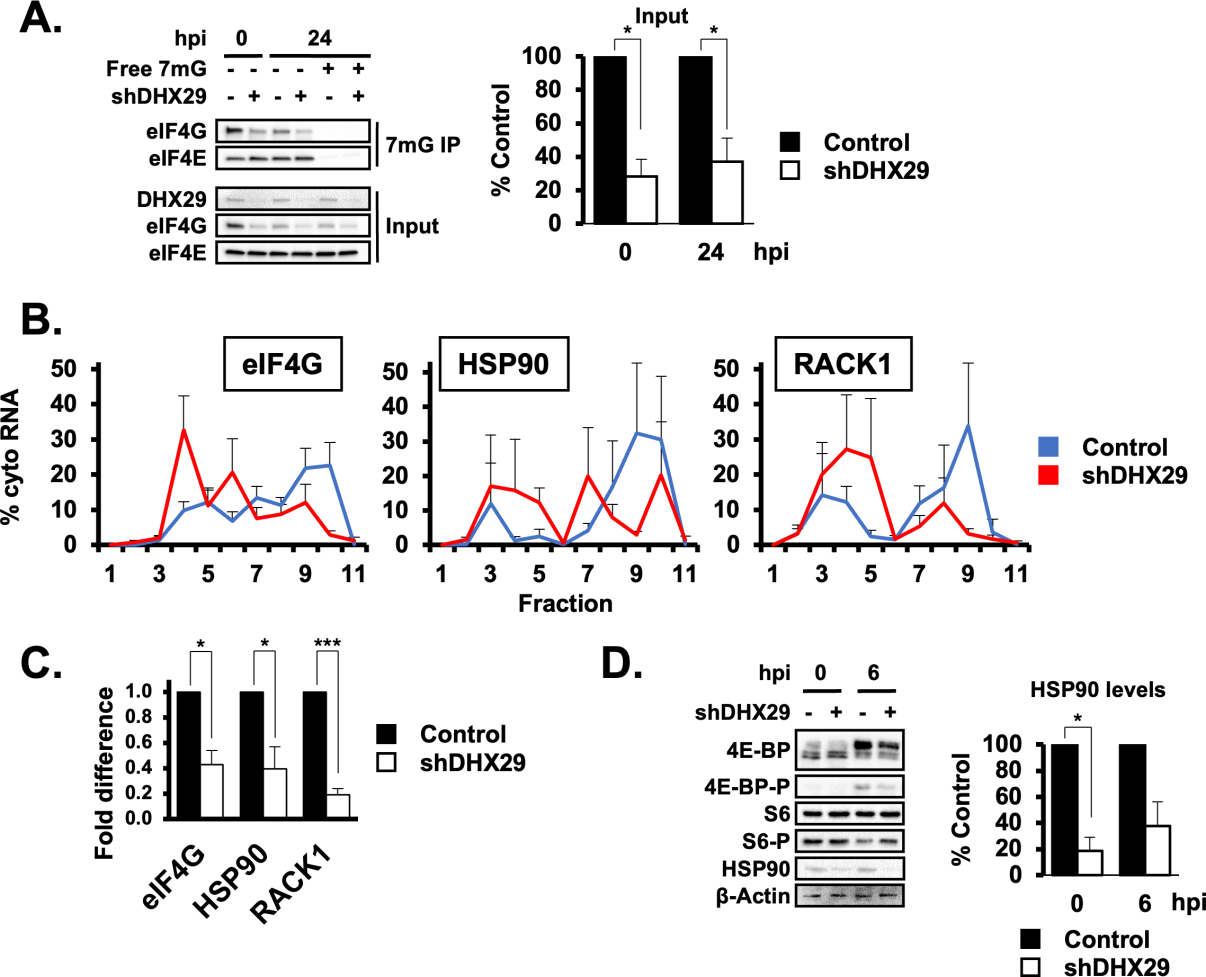
DHX29 depletion disrupts eIF4F by reducing eIF4G expression without affecting mTOR activity. (**A**) Left: human fibroblasts transduced with lentivirus expressing scrambled control or DHX29-specific shRNAs were infected with HCMV (MOI of 3). Twenty-four hours post-infection, m^7^G cap-binding complexes were precipitated from cell lysates and analyzed by Western blot. Right: densitometry of Western blot images was used to calculate the percent change in eIF4G protein levels (input) at 0 and 24 h between cells expressing control or DHX29-specific shRNAs. (**B**) Cells were treated and infected as in panel A, and cytoplasmic extracts were resolved over 10%–50% linear sucrose gradients. The percent of the eIF4G, HSP90, or actin in each gradient fraction was measured by qRT-PCR. (**C**) Graph comparing the amount of each RNA in gradient fractions containing polysomes in control or shDHX29-expressing cells. The amount of each mRNA in control cells was set to 1. (**D**) Left: Western blot showing the phosphorylation state of the mTOR substrates 4E-BP1 and rpS6 as well as HSP90 protein levels in control or DHX29-depleted cells. Right: densitometry of Western blot images was used to calculate the percent change in HSP90 protein levels at 0 and 6 h between cells expressing control or DHX29-specific shRNAs. The mean data from three independent experiments are shown (*P < 0.05; **P < 0.005; ***P < 0.001).

For some cellular mRNAs, DHX29 enhances translation in cooperation with the eIF4F translation initiation complex (7). We therefore tested the effect of DHX29 depletion on eIF4F complex formation during infection. Proteins associated with the m^7^G mRNA cap were captured from infected cell extracts using m^7^G Sepharose, and the presence of eIF4F subunits was analyzed by Western blot. While eIF4E was recovered at similar levels from control and DHX29-depleted cells, the amount of the eIF4G protein associated with the beads was reduced in the absence of DHX29 (Fig. 4A). Decreased eIF4F abundance could reflect a decrease in the activity of the mTOR kinase, which positively regulates eIF4F formation by phosphorylating the 4E-BP1 translational repressor; unphosphorylated 4E-BP1 competes with eIF4G for binding to eIF4E. However, the decrease in eIF4G binding when DHX29 was depleted was not due to decreased mTOR activity, as the levels of phosphorylated 4E-BP and rpS6 were similar to those in infected control fibroblasts (Fig. 4D). Rather, the decrease in eIF4G association with m^7^G Sepharose correlated with reduced steady-state levels of eIF4G in DHX29-depleted cells (Fig. 4A).

The eIF4G mRNA has a complex 5′ UTR (31, 32), suggesting that the defect in eIF4G protein expression in the absence of DHX29 may be due to decreased translation. Using sucrose density centrifugation, we found that DHX29 depletion resulted in a 50% reduction in the amount of eIF4G mRNA in gradient fractions containing polysomes, with a concomitant increase in the amount of eIF4G mRNA localizing to gradient fractions containing monosomes and ribosomal subunits (Fig. 4B). If DHX29 is necessary for eIF4G expression, then eIF4F-dependent mRNA translation should be reduced when DHX29 is depleted. Consistent with this hypothesis, DHX29 depletion reduced the association of the eIF4F-dependent HSP90 and RACK1 mRNAs with polysomes (Fig. 4C and reference 10) by 50% and 60%, respectively (Fig. 4D). These data show that efficient eIF4G mRNA translation, and thus eIF4F complex formation, requires DHX29 expression in HCMV-infected cells. Furthermore, these data suggest that DHX29 is necessary for the efficient translation of multiple representative eIF4F-dependent mRNAs.

### Reduced HCMV immediate-early protein synthesis is not due to reduced eIF4G expression

**Fig 5:**
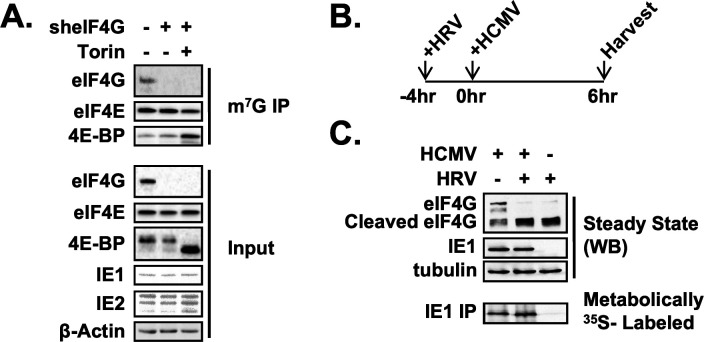
Efficient HCMV IE protein expression does not require eIF4G. (**A**) Human fibroblasts transduced with lentivirus encoding scrambled control or eIF4G-specific shRNAs were infected with HCMV. Six hours post-infection, cap-binding complexes were precipitated from cell lysates (m^7^G IP) and analyzed by Western blot along with input lysates (steady state). (**B**) Timeline of HRV and HCMV infection for experiment in panel C. (**C**) Human fibroblasts were infected with HRV, followed by HCMV infection 4 h later (top). Six hours post-infection, proteins were analyzed by Western blot or were metabolically labeled with ^35^S-methionine for 30 min before being immunoprecipitated with an anti-IE1 antibody and analyzed by gel electrophoresis and autoradiography (bottom).

We previously found that inhibiting or disrupting eIF4F does not detectably impact IE1 mRNA translation (10), suggesting that a reduction in eIF4G expression and eIF4F complex formation when DHX29 levels are reduced likely does not explain the defect in IE1 and IE2 protein expression. However, for some mRNAs, eIF4G can facilitate translation initiation independent of the eIF4F complex (22, 23). To determine if reduced eIF4G expression could explain the defect in IE1 and IE2 translation in the absence of DHX29, we measured the effect of eIF4G1 depletion on IE1 and IE2 protein levels. In addition, we treated eIF4G1-depleted cells with the mTOR inhibitor Torin1 (Fig. 5A), which inhibits eIF4G incorporation into the eIF4F complex (10, 13, 33). Torin1 treatment of eIF4G1-depleted fibroblasts enhanced 4E-BP binding to the m^7^G Sepharose as expected; however, IE1 and IE2 protein levels were unchanged by eIF4G1 depletion alone or in combination with Torin1 treatment. As an additional measure of the role of eIF4G in HCMV immediate-early expression, we measured IE1 protein levels and rates of synthesis in cells where eIF4G had been inactivated by prior infection with human rhinovirus (Fig. 5B), which encodes a protease that cleaves eIF4G, thereby disrupting its interaction with eIF4E. The steady-state protein levels of IE1 were unaffected by prior human rhinovirus (HRV) infection despite efficient eIF4G cleavage. In addition, the same amount of IE1 was synthesized during the final 30 min of the experiment in the presence or absence of HRV infection (Fig. 5C). Together, these results are consistent with the conclusion that full-length eIF4G or eIF4G cleavage products are not necessary for the efficient translation of the IE1 and IE2 mRNAs and suggest that decreased IE1 and IE2 mRNA translation in the absence of DHX29 is not the result of decreased eIF4G expression. These results are consistent with our prior studies showing that disruption of the eIF4F complex or inhibition of its components does not impact HCMV IE1 and IE2 expression (10).

## DISCUSSION

Our results reveal a novel role for DHX29 in HCMV infection. We found that DHX29 is required for efficient HCMV replication and that the association of DHX29 with the translation machinery is increased in infected cells. In the absence of DHX29, the IE1 and IE2 mRNAs are translated less efficiently. In addition, DHX29 depletion decreases the expression of the eIF4G component of the eIF4F translation initiation complex, which is required for the expression of cellular proteins necessary for efficient HCMV replication (25). However, the defect in IE1 and IE2 expression was independent of the effect on eIF4G, as both IE1 and IE2 proteins were efficiently expressed when eIF4G was depleted or inactivated, consistent with previous studies showing a minimal role for the eIF4F translation initiation complex in the translation of HCMV mRNAs. Together, these results suggest that DHX29 has two roles in HCMV replication—directly supporting the translation of viral mRNAs as well as the expression of cellular proteins needed for virus replication.

These data raise the question of why DHX29 is required for the translation of the IE1 and IE2 mRNAs. DHX29 is required for the efficient translation of mRNAs containing moderate to extensive RNA structure (Δ*G* < −40 kcal/mol) in their 5′ UTRs (24). The predominant transcripts encoding IE1 and IE2 early in infection contain a 136 nt 5′ UTR with a predicted free energy of <−40 kcal. Similarly, the 5′ UTR of transcripts encoding eIF4G1 are 368 nt in length (31, 32) and are predicted to contain highly structured regions with free energies of −100 kcal/mol (31, 34), potentially explaining the requirement for DHX29 for efficient eIF4G1 protein expression. In contrast, the actin mRNA 5′ UTR has a predicted free energy of <−16 kcal/mol and does not require DHX29 for its translation (Fig. 3 and reference 24). The nature of the 5′ UTR of the IE1 and IE2 mRNAs therefore likely explains the need for DHX29 for their translation and likely explains the role for DHX29 in eIF4G expression. While the full-length mRNA structure for most HCMV transcripts remains to be defined, the high GC content of the HCMV genome suggests DHX29 may be similarly required for the efficient translation of additional viral mRNAs as well.

Our results also raise the question of how DHX29 facilitates the translation of transcripts encoding IE1 and IE2. DHX29 is a non-processive RNA helicase whose activity is enhanced by binding to the 40S ribosomal subunit (7). DHX29 is located at the mRNA entry channel of the 48S translation initiation complex, where it interacts with helix 16 of the 18S rRNA and specific components of the eIF3 complex (6, 35). From its position in the 48S complex, DHX29 is thought to resolve RNA structures that otherwise impede mRNA transit through the entry channel of the ribosome (36). Interestingly, the 5′ UTR of mRNAs encoding IE1 and IE2 contain a stable hairpin immediately 5′ of the AUG initiation codon (14). One potential model explaining the role of DHX29 in IE1 and IE2 expression is that DHX29 associates with 48S complexes bound to mRNAs encoding IE1 and IE2 and enhances unwinding of the AUG-proximal hairpin to facilitate 48S scanning of the 5′ UTR and ensure correct recognition of the IE1 and IE2 start codon.

Our results also shed further light on the role of the eIF4F initiation complex in the translation of HCMV mRNAs. For many mRNAs, translation begins with the nucleation of the tripartite eIF4F complex on the mRNA 5′ cap. The eIF4E protein binds the cap and recruits the eIF4G scaffold protein, which recruits the 43S preinitiation complex, and the eIF4A RNA helicase, which unwinds RNA structures to enhance scanning (37). Our group and others previously found that viral mRNA translation is largely resistant to eIF4F disruption and depletion or inhibition of eIF4A (10, 11, 38). Here, we find that eIF4G abundance and functionality are also dispensable for IE1 and IE2 expression. IE1 protein synthesis was unaffected when eIF4G was depleted in HCMV-infected cells or when full-length eIF4G was inactivated by rhinovirus coinfection with HCMV. In specific instances, an eIF4G cleavage product can functionally substitute for full-length eIF4G; however, these data show that depletion of eIF4G prior to HCMV infection was similarly ineffective at reducing IE1 and IE2 expression, even when residual eIF4F complex was disrupted by treatment with the mTOR inhibitor Torin1. These data suggest that the reduced expression of eIF4G when DHX29 is depleted is unlikely to be the cause of the reduction in IE1 and IE2 expression. However, the effect on viral replication may not be inconsequential, as eIF4F plays a critical role in the translation of cellular mRNAs that HCMV requires for replication (25). These data provide further support for non-canonical translation initiation on HCMV mRNAs independent of eIF4F or its components.

Interestingly, DHX29 regulates translation initiation for other viruses as well. For example, Sindbis virus 26S RNA requires DHX29 for efficient initiation complex formation (39). Other viruses use alternatives to cap-dependent translation initiation that may also be influenced by DHX29. Small positive-stranded RNA viruses typically have structured 5′ UTRs, many of which initiate translation in a cap-independent manner via an IRES, and a subset of picornavirus IRESs require DHX29 for their activity (8, 40). Our results showing the first role for DHX29 in the translation of DNA virus mRNAs suggest that DHX29 may be a common host factor hijacked by viruses more generally, suggesting a potential role for DHX29 inhibitors as broad-spectrum antiviral drugs.

## SUPPLEMENTAL MATERIAL

Figure S1 mbio.03542-25-s0001.tiff Data using an additional DHX29-specific shRNA to validate key results.

ASM does not own the copyrights to Supplemental Material that may be linked to, or accessed through, an article. The authors have granted ASM a non-exclusive, world-wide license to publish the Supplemental Material files. Please contact the corresponding author directly for reuse.
