# Supplementary figures and images for: The host DHX29 RNA helicase regulates HCMV immediate-early protein synthesis
Source: mBio. 2026 Jun 9;17(7):e03542-25. doi: 10.1128/mbio.03542-25 (PMC13343918; doi:10.1128/mbio.03542-25)

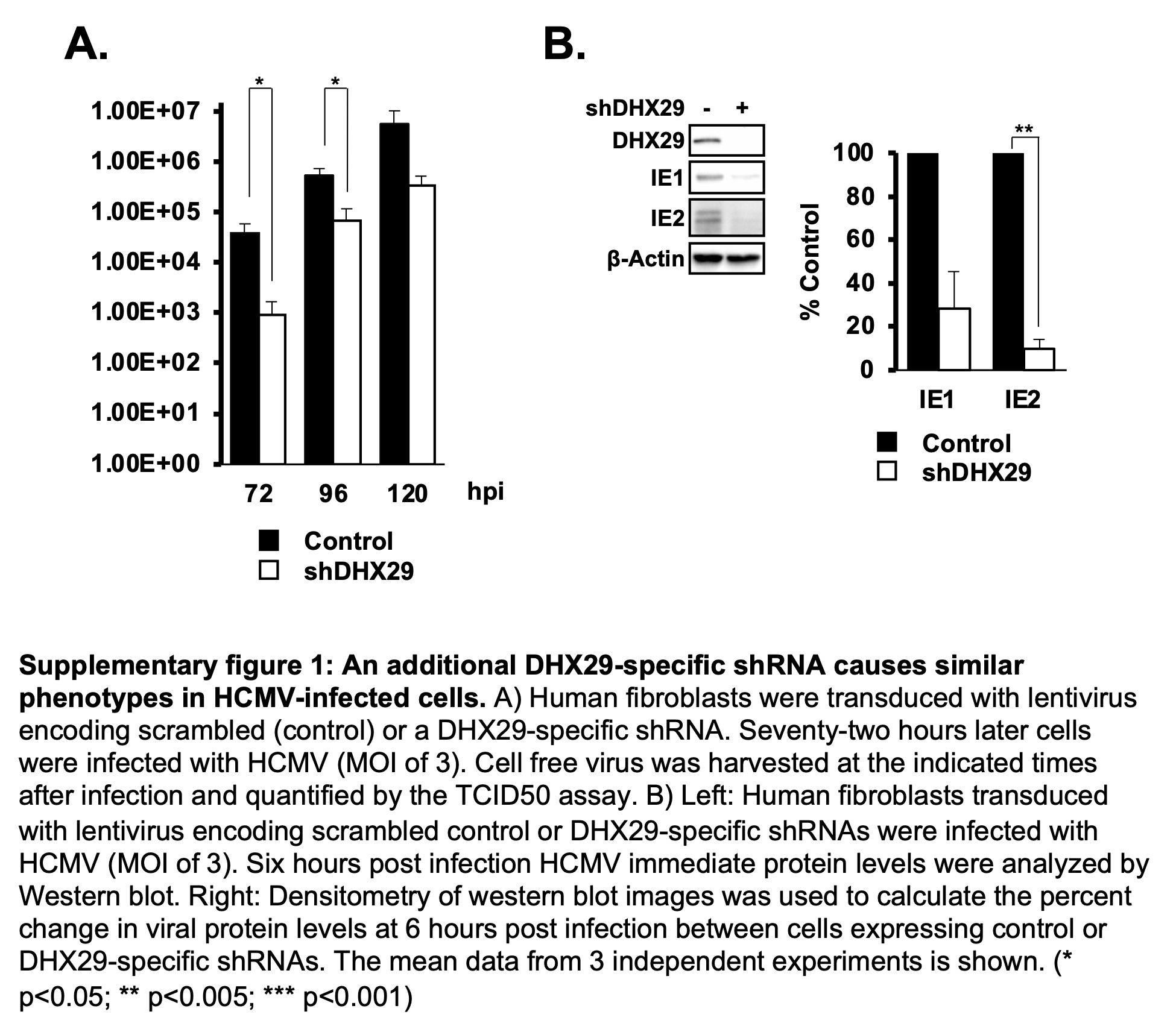

Supplement: Figure S1 — Data using an additional DHX29-specific shRNA to validate key results. [file mbio.03542-25-s0001.tiff]
